# 64 Worse Itch and Fatigue in Racial and Ethnic Minorities: A Burn Model System Cross-sectional Study

**DOI:** 10.1093/jbcr/irad045.038

**Published:** 2023-05-15

**Authors:** Paul Won, Sarah Stoycos, Li Ding, Kara McMullen, Karen Kowalske, Barclay Stewart, Haig Yenikomshian

**Affiliations:** University of Southern California, Pasadena, California; VA National Center for PTSD and Boston University School of Medicine, Jamaica Plain, Massachusetts; University of Southern California, Los Angeles, California; University of Washington, Seattle, Washington; UT Southwestern Medical Center, Dallas, Dallas, Texas; University of Washington, Seattle, Washington; University of Southern California, Los Angeles, California; University of Southern California, Pasadena, California; VA National Center for PTSD and Boston University School of Medicine, Jamaica Plain, Massachusetts; University of Southern California, Los Angeles, California; University of Washington, Seattle, Washington; UT Southwestern Medical Center, Dallas, Dallas, Texas; University of Washington, Seattle, Washington; University of Southern California, Los Angeles, California; University of Southern California, Pasadena, California; VA National Center for PTSD and Boston University School of Medicine, Jamaica Plain, Massachusetts; University of Southern California, Los Angeles, California; University of Washington, Seattle, Washington; UT Southwestern Medical Center, Dallas, Dallas, Texas; University of Washington, Seattle, Washington; University of Southern California, Los Angeles, California; University of Southern California, Pasadena, California; VA National Center for PTSD and Boston University School of Medicine, Jamaica Plain, Massachusetts; University of Southern California, Los Angeles, California; University of Washington, Seattle, Washington; UT Southwestern Medical Center, Dallas, Dallas, Texas; University of Washington, Seattle, Washington; University of Southern California, Los Angeles, California; University of Southern California, Pasadena, California; VA National Center for PTSD and Boston University School of Medicine, Jamaica Plain, Massachusetts; University of Southern California, Los Angeles, California; University of Washington, Seattle, Washington; UT Southwestern Medical Center, Dallas, Dallas, Texas; University of Washington, Seattle, Washington; University of Southern California, Los Angeles, California; University of Southern California, Pasadena, California; VA National Center for PTSD and Boston University School of Medicine, Jamaica Plain, Massachusetts; University of Southern California, Los Angeles, California; University of Washington, Seattle, Washington; UT Southwestern Medical Center, Dallas, Dallas, Texas; University of Washington, Seattle, Washington; University of Southern California, Los Angeles, California; University of Southern California, Pasadena, California; VA National Center for PTSD and Boston University School of Medicine, Jamaica Plain, Massachusetts; University of Southern California, Los Angeles, California; University of Washington, Seattle, Washington; UT Southwestern Medical Center, Dallas, Dallas, Texas; University of Washington, Seattle, Washington; University of Southern California, Los Angeles, California

## Abstract

**Introduction:**

Racial and ethnic minority patients may experience worse hypertrophic scar after burn injury than Non-Hispanic, White patients. Consequently, they may encounter differences in scar-related recovery domains including itch and fatigue. We examined disparities in post-burn injury itch and fatigue in minority patients to better inform counseling and treatment considerations.

**Methods:**

From the multicenter National Institute of Disability, Independent Living and Rehabilitation Research Burn Model System Database (2015-2019), we analyzed outcomes at three time-points (discharge from index hospitalization, 6- and 12-months post-injury) using the 5-D Itch and PROMIS-29 Fatigue measures. Multi-level linear mixed effects regression modeling analyzed associations between race/ethnicities and outcomes over time.

**Results:**

Of 893 total patients, 149 (17%) patients were Hispanic/Latino, 603 (68%) Non-Hispanic, White, and 86 (10%) Black. Minority patients reported higher/worse itch scores at all time points compared to Non-Hispanic, White patients. Itch scores were significantly higher for Black patients at 6 months (β=1.42, *p=0*.03) and 12 months (β=3.36, *p*< 0.0001) when compared to Non-Hispanic, White patients. Black patients reported higher fatigue scores than Non-Hispanic, White patients at all time points. Fatigue scores were significantly higher for Hispanic/Latino patients at discharge (β=6.17, *p*< 0.0001), 6 months (β=4.49, *p*=0.0003), and 12 months (β=6.27, *p*< 0.0001) than Non-Hispanic, White patients.

**Conclusions:**

This study supports investigation of potential factors leading to increased itch and fatigue such as genomic scar differences, relative treatment effects (e.g., medications, cognitive processing therapy), and psychosocial impacts of these symptoms.

**Applicability of Research to Practice:**

In the short-term, minority patients may benefit from additional counseling and focused treatments addressing itch and fatigue after burn injury.

